# Performance of Large Language Models in Oral Health Consultations and the Consistency of the ‘AI-as-a-Judge’ Framework

**DOI:** 10.1016/j.identj.2026.109597

**Published:** 2026-04-23

**Authors:** Weibin Shen, Teng Zhang, Yaqi Liu

**Affiliations:** a Key Laboratory of Shaanxi Province for Craniofacial Precision Medicine Research, College of Stomatology, Xi’an Jiaotong University, Xi’an, Shaanxi, China; b Clinical Research Center of Shaanxi Province for Dental and Maxillofacial Diseases, College of Stomatology, Xi’an Jiaotong University, Xi’an, Shaanxi, China; c College of Stomatology, Xi’an Jiaotong University, Xi’an, Shaanxi, China; d BYD Group Digitalization Center, Xi’an, Shaanxi, China; e Department of Oral Public Health, The Affiliated Stomatological Hospital of Xuzhou Medical University, Xu Zhou, Jiangsu, China

**Keywords:** Artificial intelligence, Large language models, Artificial Intelligence-as-a-Judge, Oral health, Human–Artificial Intelligence consistency

## Abstract

**Objective:**

To evaluate the performance of large language models (LLMs) in responding to oral health consultations and to examine the consistency between the AI-as-a-Judge evaluation framework and human expert ratings.

**Methods:**

Nine oral health questions were selected from the World Dental Federation (FDI) official website and posed to 6 models: GPT-5.0, Gemini-3.0, DeepSeek-V3, Qwen3-Max, Kimi-K2 and Doubao-1.8-Pro. Responses were independently scored by 2 clinicians and 3 AI judges.

**Results:**

Significant performance differences were observed among the 6 models, with DeepSeek-V3 and Doubao-1.8-Pro achieving the best results. Inter-rater consistency among human experts was good (ICC = 0.860), while consistency among AI judges was low (ICC = 0.538). Human–AI consistency was extremely low (ICC = 0.215) and AI judges exhibited a significantly stricter scoring tendency.

**Conclusion:**

Leading domestic LLMs have attained competitive performance in oral health consultations. However, the current ‘AI-as-a-Judge’ framework demonstrates significant inconsistency and bias compared to human experts, suggesting that automated AI evaluation systems are not yet a reliable substitute for human expert review in clinical contexts.

## Introduction

Artificial intelligence (AI), as a technical science that simulates and extends human intelligence,^1^ has seen its subfield – natural language processing (NLP) – evolve to the stage of large language models (LLMs). Models represented by DeepSeek and GPT, trained on massive datasets, can understand and generate coherent text,^2^^,^^3^ profoundly transforming interaction paradigms in the medical field.^4^^,^^5^ In dentistry, the integration of AI has been comprehensively reviewed, highlighting both its transformative potential and inherent challenges.^6^^,^^7^

Building upon this foundation, LLMs are increasingly being applied across various dental domains.^8^ Research indicates that GPT can assist in diagnosing oral diseases through medical image analysis, enabling personalised treatment,^9^ and exhibits higher sensitivity in detecting enamel lesions, though with a noted risk of overtreatment.^10^ Beyond clinical applications, LLMs have shown potential in optimising hospital workflows.^11^ However, existing studies predominantly focus on the English context, leaving the evaluation framework for Chinese-language LLMs in specialised fields like dentistry underdeveloped. While recent investigations have begun to assess LLM performance in specific oral conditions such as HIV-associated Kaposi sarcoma,^12^ these evaluations remain largely confined to English-language contexts and do not address the reliability of using LLMs as evaluative agents in clinical settings. This gap is particularly significant given that the current LLM landscape is now characterised by both international models (eg, GPT, Gemini) and rapidly advancing domestic Chinese models. Meanwhile, domestic Chinese models such as DeepSeek, Doubao, Kimi and Qwen have increasingly emerged as promising tools in medical question–answering scenarios, leveraging deep optimisation of Chinese corpora and adaptation to localised application contexts. For instance, as described on its official website, DeepSeek-V3 is positioned as a specialised model for complex logical reasoning, focusing on intricate reasoning tasks. In contrast, Qwen integrates multimodal input capabilities, enabling it to process not only textual data but also various other data types, including images and audio. These growing capabilities of these models necessitate the development of a dedicated evaluation framework tailored to specialised, high-stakes fields like dentistry.

Furthermore, while prior research has largely emphasised clinical diagnosis and treatment, there is a lack of systematic assessment regarding the reliability of oral health consultations obtained via AI applications, particularly when patients cannot promptly contact a physician. These recent studies evaluating LLM performance in specific oral conditions exemplify the growing interest in this area, yet they remain confined to disease-specific contexts and do not address the patient consultation scenario or the reliability of using LLMs as evaluative agents. Additionally, with the rise of the ‘AI-as-a-Judge’ paradigm – where LLMs are employed to evaluate the outputs of other LLMs – recent literature has documented the increasing adoption of LLMs as automated evaluators in fields ranging from education to software engineering and this paradigm is now extending into healthcare.^13^ There is a pressing need to scrutinise the credibility of this automated evaluation method. This scrutiny is especially critical in high-risk medical domains, where the validity of an AI-generated evaluation could have downstream implications for clinical safety. As these AI tools become increasingly accessible to patients and are being integrated into clinical workflows, the urgency of establishing validated evaluation frameworks has never been greater. To address these gaps, this study evaluates the performance of 6 LLMs (including both international and leading domestic models) in oral health consultations, aiming to provide a basis for clinicians to guide patients in the rational use of AI tools. Simultaneously, by comparing the discrepancies between AI and expert human evaluations, it further explores the capabilities and limitations of AI in the governance of oral health information.

## Methods

### Study design

This study employed a cross-sectional analytical design to evaluate and compare the performance of 6 LLMs in responding to oral health consultation questions, using expert human evaluations as the gold standard. A secondary objective was to assess the reliability and validity of an ‘AI-as-a-Judge’ framework, wherein 3 additional LLMs were employed to evaluate the same responses. The study further examined the temporal stability of model responses by conducting 2 rounds of data collection separated by a 15-day interval.

### Study subjects and ethical approval

This study selected 6 mainstream LLMs – GPT-5.0, Gemini-3.0, DeepSeek-V3, Qwen3-Max, Kimi-K2 and DouBao-1.8-Pro – conducting question-and-answer sessions on their official websites. The collected responses were recorded using Microsoft Excel (Microsoft Corporation). This study was conducted in accordance with the local ethical framework and regulations described in the Declaration of Helsinki. As it did not involve human or animal experiments, no additional ethical approval was required.

### Evaluators

Two senior clinicians (the authors) from the affiliated stomatological hospitals of Xi’an Jiaotong University and Xuzhou Medical University, each with over 8 years of clinical experience in dentistry, served as the human expert evaluators. An independent arbitrator, a senior researcher with a dental background who was not involved in the authorship of this study, was appointed to resolve scoring discrepancies. The AI judge panel consisted of 3 leading general-purpose LLMs: GLM-4.7, Tencent HY 2.0 and Baichuan-M3.

### Question selection and data collection

Nine questions related to the field of oral health (Table 1) were selected from the World Dental Federation (FDI) website.^14^ These questions cover a range of topics, including paediatric oral care, geriatric oral health, pregnancy-related oral health and disease prevention. The original English questions were translated into Chinese by a bilingual dental expert and reviewed by a second expert to ensure accuracy and cultural appropriateness. The FDI website answers were used as a reference for the experts during their evaluation but did not constitute the direct scoring criterion. All models were prompted in Chinese using a zero-shot approach (ie, the question was directly input without any additional prompt engineering). This approach was chosen to standardise the testing conditions across all models and to assess baseline performance without the influence of engineered prompts.

**Table 1:** Oral health-related questions from the World Dental Federation (FDI).

Question ID	Question content
1.	How to clean a baby’s mouth?
2.	Bottle-feeding tips and pacifiers
3.	How should elderly individuals with dry mouth perform oral care?
4.	How can I maintain oral health during pregnancy?
5.	What can you do to prevent oral and dental trauma?
6.	How to keep your mouth healthy throughout life?
7.	Why does oral health matter?
8.	What are the main risk factors for oral diseases?
9.	How many people are affected by oral diseases?

To assess the temporal stability of the responses, the same questions were posed again after a 15-day interval (first round: October 1, 2025; second round: October 15, 2025). This interval was chosen to minimise the risk of recall bias from the human experts during the second round of evaluation, while being long enough to potentially capture any significant updates or variability in the LLMs’ outputs. All interactions were conducted using the publicly available web interfaces of the models with their default parameter settings (eg, temperature = 0) to ensure reproducibility. The responses were recorded verbatim in Microsoft Excel. To minimise recall bias, the 2 clinicians were blinded to the model identity and the round of response generation during the evaluation process.

### Evaluation criteria and scoring process

The collected responses were randomly numbered and divided into 2 sets. A standardised evaluation rubric was developed, comprising 5 dimensions: scientific accuracy, logical rigour, clinical practicality, terminology accuracy and answer completeness (Table 2). Each dimension was scored on a 5-point Likert scale (1 = incorrect to 5 = excellent).

**Table 2:** Evaluation criteria for oral health-related questions.

Evaluation dimension	5 points (excellent)	4 points (good)	3 points (satisfactory)	2 points (poor)	1 point (incorrect)
Scientific accuracy	Fully consistent with scientific evidence	Largely consistent with scientific evidence	Generally accurate, with minor errors	Contains obvious factual medical errors	Completely contradicts scientific evidence
Logical rigour	Information is accurate and complete	Information is mostly accurate	Information is generally accurate, with minor detail errors	Logic is confusing, missing key reasoning steps	Logically flawed, completely off-topic
Clinical practicality	Recommendations are specific and align with guidelines	Recommendations are feasible and largely align with guidelines	Recommendations are reasonable but lack detail	Impractical and difficult to implement	Completely detached from clinical practice
Terminology accuracy	Terminology is professional, clear and unambiguous	Terminology is mostly standard	Mostly correct terminology, with occasional vagueness	Partially incorrect terminology, potentially misleading	Severe errors in terminology usage
Answer completeness	Covers all key points comprehensively	Nearly comprehensive, missing only minor details	Covers most key points, but omits some	Misses most key points	Severely lacks core information

Prior to the formal scoring, the 2 clinician evaluators participated in a calibration session using a sample of responses randomly selected from the total pool, which were excluded from the final statistical analysis. This session aimed to ensure a shared understanding of the 5 evaluation dimensions and to standardise the application of the scoring criteria.

Subsequently, they independently scored all responses, with the average score taken as the final score for each response.

Scoring and Adjudication: For responses with a scoring discrepancy greater than 20% (ie, a mean score difference >1 point between the 2 clinicians), the independent arbitrating expert reviewed the response and the original scores to render a final, binding decision.

Subsequently, the AI judge panel consisting of 3 large models (GLM-4.7, Tencent HY 2.0 and Baichuan-M3) scored the same responses according to the same criteria. All scorers (both human and AI) were assigned the role of ‘oral medicine expert’ via system prompts to ensure consistency in the evaluation perspective.

### Validity and reliability

The inter-rater reliability between the 2 human experts was assessed using the intraclass correlation coefficient (ICC). The internal consistency of the AI judge panel was similarly evaluated using ICC. To assess the validity of the AI-as-a-Judge framework, human–AI consistency was calculated using ICC and visualised with Bland–Altman plots.

### Data processing and statistical analysis

Data coding, input and cleaning were performed using Microsoft Excel. Statistical analyses were performed using SPSS version 22 (IBM Corp.) and R version 4.2.1 (R Foundation for Statistical Computing). Normality of the data was assessed using the Shapiro–Wilk test and Q–Q plots. The Friedman test was used to compare overall differences among the 6 models; for significant results, post hoc pairwise comparisons were conducted using the Conover test with Holm–Bonferroni correction. Intraclass correlation coefficients (ICC [2, 1] and ICC [2, k]) were employed to evaluate inter-rater reliability and human–AI consistency, respectively. Bland–Altman plots were constructed to calculate bias and 95% limits of agreement (LoA), supplemented by paired *t* tests to assess systematic bias. The stability of responses between the 2 rounds was analysed using the Wilcoxon signed-rank test. A linear mixed model (LMM) was constructed for attribution analysis of influencing factors, with question ID included as a random effect to account for variations in question difficulty. All tests were 2-tailed, with a significance level of *α* = 0.05.

## Results

### Overall score performance of models

**Fig. 1:**
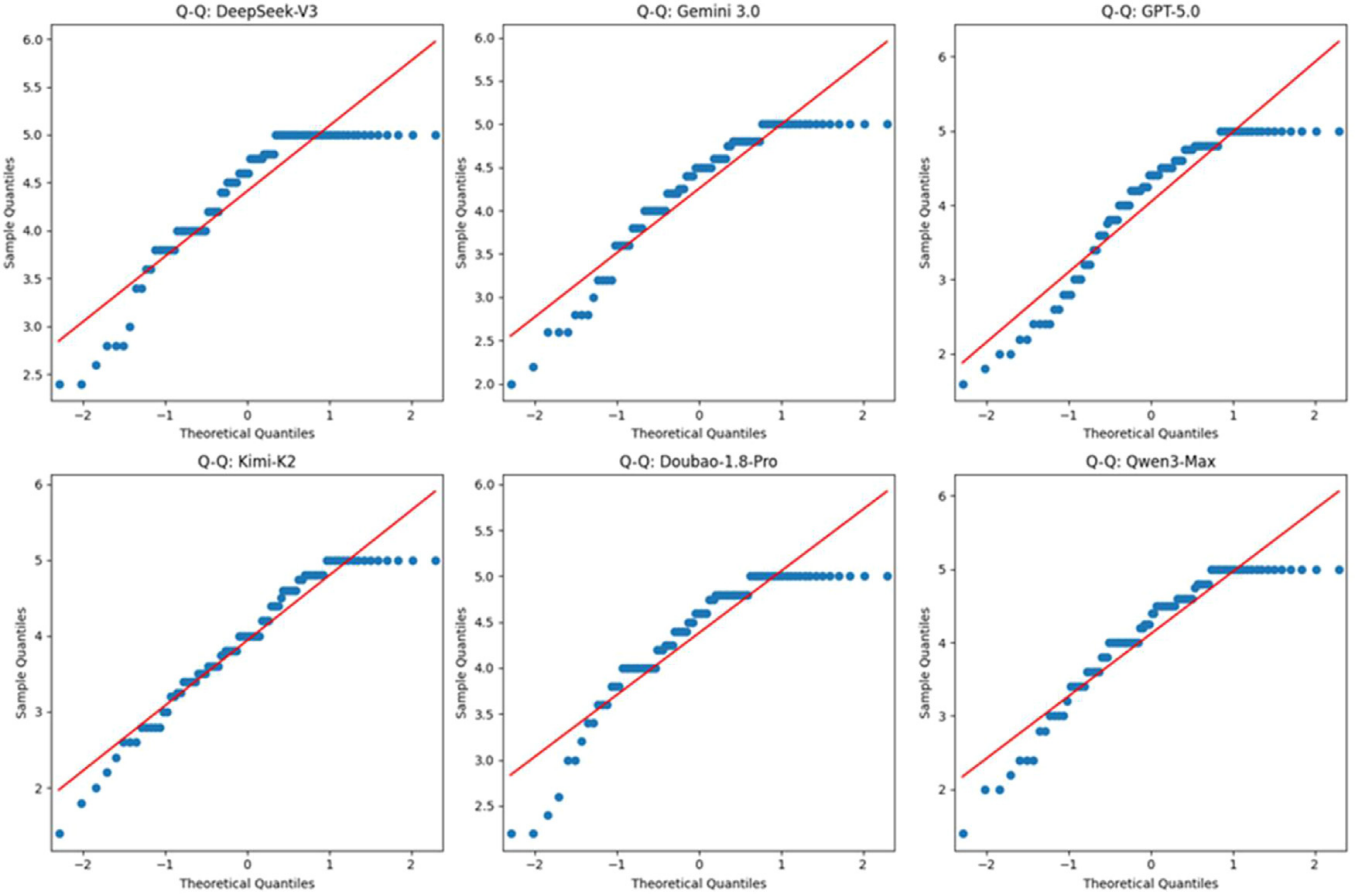
Q–Q plot for normality of distribution.

A total of 540 valid scoring samples were included in this study (9 questions × 2 rounds × 6 models × 5 judges). The Shapiro–Wilk test indicated that the scores for each model did not follow a normal distribution (all *P* < .001) and the Q–Q plots (Figure 1) also showed data points deviating from the reference line. Therefore, nonparametric tests were employed and results are presented as median (interquartile range) [M (IQR)].

In the first round of evaluation, Doubao-1.8-Pro [4.80 (1.00)], Gemini 3.0 [4.60 (1.00) and DeepSeek-V3 [4.40 (1.00)] achieved relatively high scores, while Kimi-K2 scored relatively lower [3.80 (1.40)]. In the second round of evaluation, most models showed minor fluctuations in scores: DeepSeek-V3 increased to 4.75 (0.60), Kimi-K2 increased to 4.20 (1.20) and GPT-5.0 decreased slightly to 4.25 (1.35) (Table 3).

**Table 3:** Scores of each model in the 2 rounds of evaluation.

LLM	Round	Mean	SD	Median	IQR
DeepSeek-V3	R1	4.28	0.72	4.40	1.00
	R2	4.53	0.63	4.75	0.60
Doubao-1.8-Pro	R1	4.43	0.70	4.80	1.00
	R2	4.33	0.66	4.40	0.80
Gemini 3.0	R1	4.33	0.78	4.60	1.00
	R2	4.17	0.72	4.25	0.80
Qwen3-Max	R1	4.00	0.93	4.00	1.40
	R2	4.24	0.77	4.50	0.80
Kimi-K2	R1	3.76	0.89	3.80	1.40
	R2	4.12	0.81	4.20	1.20
GPT-5.0	R1	4.10	0.91	4.40	1.20
	R2	4.00	1.00	4.25	1.35

IQR, interquartile range; LLM, large language model; SD, standard deviation.

### Comparative analysis of performance among models

**Fig. 2:**
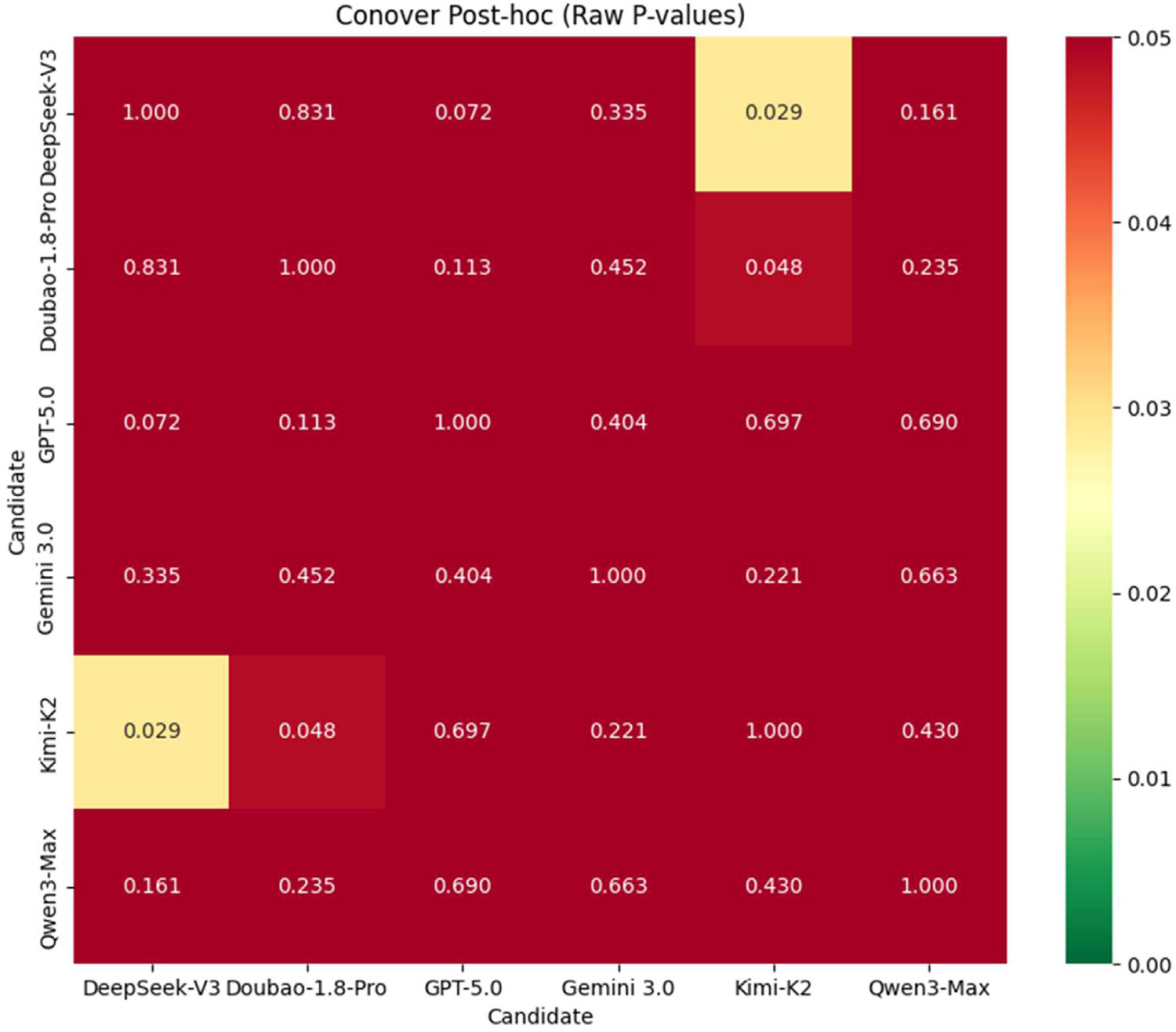
Heatmap of Conover post hoc test results.

The Friedman test revealed a significant difference in the performance of the 6 models in oral health consultations (*χ*^2^ = 29.069, *P* < .001). The mean ranks for each model were as follows: DeepSeek-V3 (4.07), Doubao-1.8-Pro (3.98), Gemini 3.0 (3.24), Qwen3-Max (3.13), GPT-5.0 (2.98) and Kimi-K2 (2.60). Conover post hoc tests (with Holm–Bonferroni correction) indicated (Figure 2) that the differences between DeepSeek-V3 and GPT-5.0 (*P* = .072) and between Doubao-1.8-Pro and GPT-5.0 (*P* = .100) approached significance, suggesting a trend towards leading domestic models outperforming GPT-5.0. No significant differences were found in any other pairwise comparisons between models (*P* > .05).

### Stability analysis of model responses

**Fig. 3:**
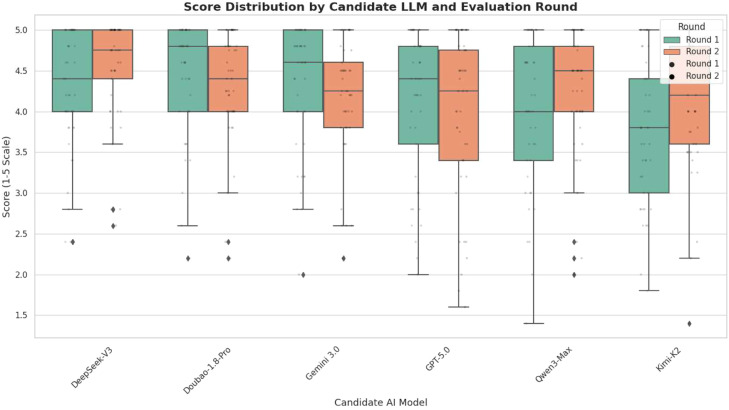
Box plot of scores for each model across 2 evaluation rounds.

The Wilcoxon signed-rank test showed no significant difference in the overall scores of the 6 models between the 2 rounds of responses (*P* = .452), indicating stable performance. The box plot (Figure 3) visually displays the score distribution of each model, with most models remaining stable across the 2 evaluation rounds. Among them, DeepSeek-V3 and Doubao-1.8-Pro demonstrated the best consistency.

### Rater consistency analysis

#### Inter-rater consistency among human experts

Following calibration, the 2 human experts demonstrated good scoring consistency: ICC (2, 1) = 0.754 (95% CI: 0.66-0.83), ICC (2, k) = 0.860, indicating excellent reliability. This provided a robust ‘gold standard’ for this study.

#### Inter-rater consistency among ai judges

The AI judge panel, consisting of 3 models (GLM-4.7, Tencent HY 2.0 and Baichuan-M3), exhibited low internal reliability: ICC (2, 1) = 0.280, ICC (2, k) = 0.538. This indicates significant divergence in the scoring logic among different AI models. Although aggregating scores from multiple models improved reliability to 0.538, this still falls considerably short of the >0.75 reliability level required to substitute for human expert consensus.

#### Human–AI consistency

**Fig. 4:**
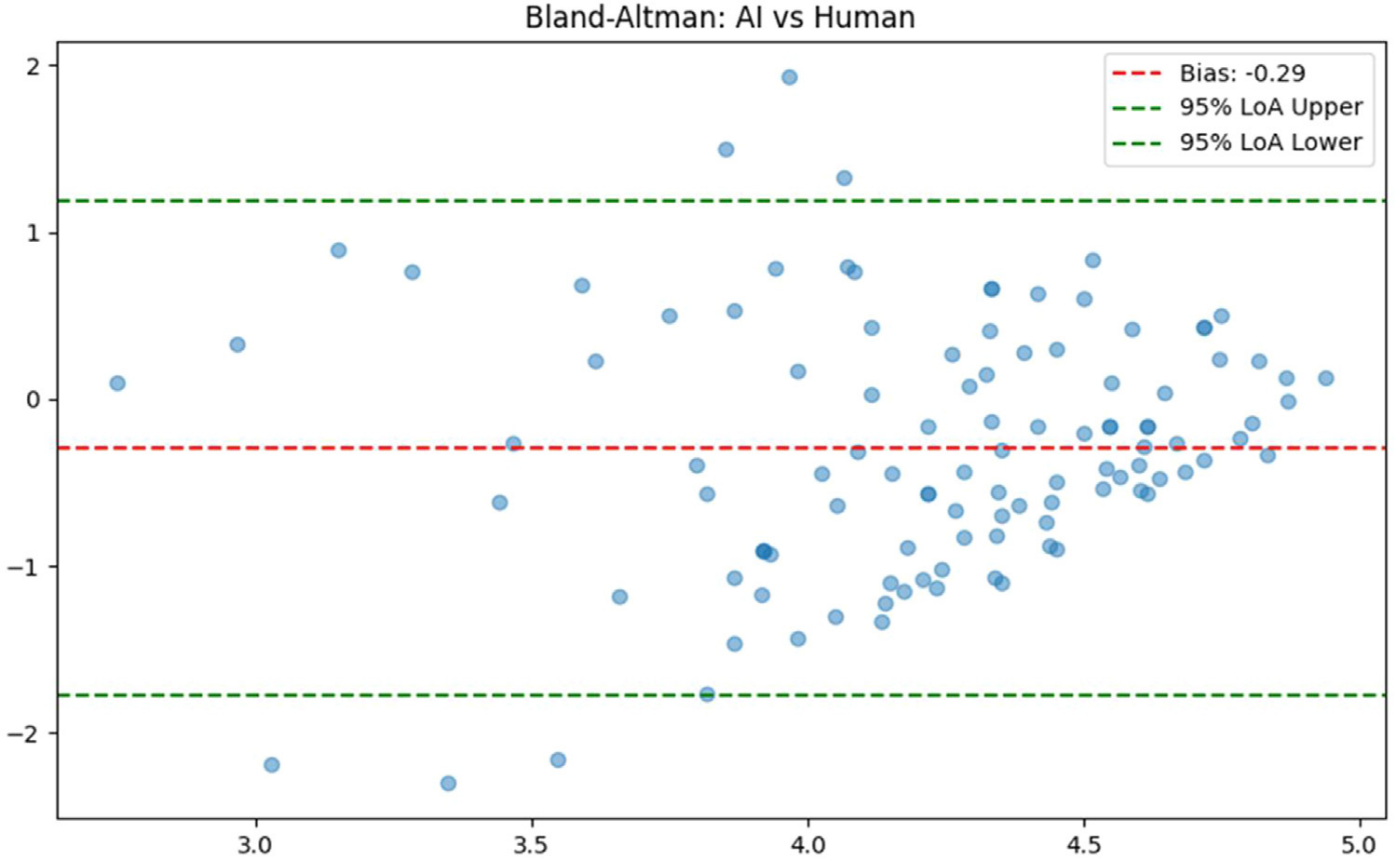
Bland–Altman plot for human-AI score consistency.

Using the mean score of the 3 AI judges as the panel’s collective judgement, the consistency analysis with the mean human expert score showed: ICC (2, 1) = 0.121 (95% CI: –0.05 to 0.29), ICC (2, k) = 0.215. Bland–Altman analysis (Figure 4) revealed a bias of –0.387 (paired *t* test, *P* < .001), indicating that AI scores were, on average, 0.39 points lower than human scores, reflecting a more stringent evaluation. The 95% limits of agreement (LoA) ranged from –1.69 to 0.91, suggesting substantial individual discrepancies between AI and human judgements on specific cases, with potential extreme outliers for some responses.

### Attribution analysis of influencing factors

The linear mixed model (LMM) was constructed with question ID as a random effect to examine the impact of model type and evaluation round on scores. The results showed that model type was a core variable influencing scores (*P* < .001): using GPT-5.0 as the reference category, both DeepSeek-V3 and Doubao-1.8-Pro exhibited significant positive effects, consistent with the findings from nonparametric tests. Evaluation round had no significant effect (*P* = .452), indicating good reproducibility of model performance. The random effects revealed some variation in difficulty across questions (Group Var = 0.101), suggesting slight fluctuations in model proficiency across different oral health subspecialty topics.

### Identifying clinical risk blind spots in automated AI evaluation

Combining the wide limits of agreement (−1.69 to 0.91) revealed by the Bland-Altman analysis with the low rater consistency indicated by ICC, this study further conducted an exploratory analysis of responses judged by human experts as having ‘clinical limitations’ (scores <4.0). Although AI judges exhibited more stringent overall scoring (bias: −0.39), the wide limits of agreement suggest significant discrepancies between human and AI judgements in specific high-risk cases. Analysis of extreme cases (score differences exceeding 1 standard deviation) revealed that disagreements were concentrated in the domains of preventive medicine consultation and safety considerations for special populations, indicating limitations in the current AI-as-a-Judge framework regarding the identification of critical information omissions with clinical implications.

## Discussion

This study systematically evaluated the performance of 6 mainstream LLMs in oral health consultations and, for the first time, examined the reliability and validity of the AI-as-a-Judge framework based on multimodel consensus. The main findings include: leading domestic models (DeepSeek-V3, Doubao-1.8-Pro) have demonstrated the ability to compete with top-tier international models in the dental domain, yet significant heterogeneity exists among models, suggesting that the reliability of AI outputs is highly dependent on specific model architectures and training strategies. This heterogeneity likely stems from differences in model architectures, training data and optimisation strategies. The strong performance of DeepSeek-V3 and Doubao-1.8-Pro in this Chinese-language context may be attributed to their extensive pretraining on high-quality Chinese corpora, including medical literature, which allows them to better understand and address the nuances of local health inquiries.

However, the core findings point to deep-seated flaws within the AI evaluation system itself. The consistency among human expert ratings was robust (ICC = 0.754), but the AI judge panel exhibited extremely low internal consistency (ICC = 0.280) and a systematic bias compared to human expert scores (mean bias –0.387, *P* < .001). This systematic bias suggests that current LLM-based evaluators may prioritise linguistic fluency and surface-level coherence over deep clinical reasoning and risk assessment. They often struggle to weigh the relative importance of clinical information, potentially penalising responses for stylistic choices while overlooking critical omissions that would be immediately apparent to a human expert. The low inter-AI consistency (ICC = 0.280) further reveals that these models have not yet converged on a stable ‘evaluation logic’, reflecting fundamental differences in their internal knowledge representation and value alignment. This interpretation aligns with a growing body of evidence regarding the limitations of LLMs. Previous studies have noted the limited deep reasoning capabilities of LLMs,^15^ with their responses relying more on pattern recognition in text than on independent scientific reasoning.^16^ This study further demonstrates that this limitation is amplified in evaluation systems cantered on LLMs.

Therefore, this study provides reference implications for the application and evaluation of AI in dentistry. First, it calls into question the feasibility of fully relying on AI to assess clinical quality and safety operations. The poor internal consistency among AI judges and their systematic bias relative to human experts collectively indicate that current evaluation frameworks based on general-purpose LLMs are not yet capable of reliably identifying professional deficiencies that are obvious to human experts. In matters of clinical safety, AI cannot currently replace human experts for final review.^17^ Second, it points the way for the future development of reliable medical AI: efforts should be directed towards constructing specialised evaluation modules that deeply integrate clinical knowledge, safety rule bases and evidence-based medicine. The focus of evaluation should shift from ‘whether the language is fluent’ to ‘whether the medical facts are correct and whether patient risks are controllable’.

This study has several limitations. First, although the evaluation questions are representative and sourced from the authoritative FDI website, their number is limited (*n* = 9). While this allowed for an in-depth, granular analysis by human experts, it restricts the generalisability of the findings across the vast breadth of oral health topics. This study should be viewed as an exploratory investigation into the validity of the evaluation framework itself. Second, the AI judge panel consisted of general-purpose models; subsequent research could compare the performance of models fine-tuned with specialised domain data as judges. Third, the study employed a zero-shot prompting strategy. Given that LLM performance can be enhanced with prompt engineering, the absence of such techniques may have limited the observed performance of the models. Therefore, the results should be interpreted as baseline performance under minimal guidance conditions. Fourth, while the human experts were calibrated and blinded, we acknowledge a potential cultural and linguistic bias, as the expert panel consisted of Chinese clinicians. This might theoretically favour models trained predominantly on Chinese corpora. However, the strong performance of international models like Gemini in certain aspects suggests that the scoring was primarily driven by clinical and scientific merit, mitigating this concern to some extent. Finally, the low internal consistency among AI judges further undermines the credibility of any single automated score, reinforcing our primary conclusion.

## Future directions and recommendations

This study highlights several key areas for future development. First, there is a clear need to move beyond general-purpose LLMs as judges and develop specialised evaluation modules that are fine-tuned on domain-specific clinical data, safety rule bases and evidence-based guidelines. Second, future research should expand the question set to include more complex scenarios requiring differential diagnosis, treatment planning and ethical judgement, rather than just factual recall.^18^ Third, given the potential for cultural and linguistic bias, international collaborative studies involving multilingual and multicenter expert panels are essential to validate findings. Fourth, building upon the zero-shot limitation acknowledged above, future research should systematically investigate the impact of various prompt engineering strategies (eg, chain-of-thought, few-shot learning, role-playing prompts) on both response generation and evaluation in oral health contexts. Fifth, rather than viewing AI as a replacement for human oversight, efforts should focus on establishing human–AI collaboration frameworks where AI serves as a first-pass screener or a provider of supporting evidence, with ultimate clinical authority remaining with human experts. For policymakers and clinicians, these findings serve as a caution that while AI tools are powerful, their outputs and evaluations must be interpreted with care, particularly in patient-facing applications. For patients and the public, these findings serve as a reminder to exercise caution when using AI tools for health-related inquiries and to discuss AI-generated information with qualified healthcare professionals before making clinical decisions.

## Conclusion

In summary, domestic LLMs such as DeepSeek-V3 and Doubao-1.8-Pro perform well in providing standardised oral health information. However, the automated AI evaluation framework suffers from dual deficiencies: low reliability (poor internal consistency) and low validity (systematic bias compared to human experts), rendering it unsuitable for the role of a clinical safety arbiter. Only by prioritising clinical safety and fostering human–AI collaboration can technological innovation be robustly implemented in the health domain.

## Author contributions

*Conceptualisation*: Shen and Zhang. *Formal analysis*: Shen, Zhang and Liu. *Methodology*: Shen, Zhang and Liu. *Writing – original*: Weibin Shen, Teng Zhang. *Writing – review and editing*: Shen, Zhang and Liu.

## Ethics statement

This study was conducted in accordance with the local ethical framework and regulations described in the Declaration of Helsinki. As it did not involve human or animal experiments, no additional ethical approval was required.

## Data availability

The datasets used and/or analysed during the current study are available from the corresponding author on reasonable request.

## Funding

This research did not receive any specific grant from funding agencies in the public, commercial, or not-for-profit sectors.

## Conflict of interest

The authors declare no competing interests.
